# Enhanced ultraviolet absorption in graphene by aluminum and magnesium hole-arrays

**DOI:** 10.1038/s41598-021-87868-7

**Published:** 2021-04-19

**Authors:** Xueling Cheng, Yunshan Wang

**Affiliations:** 1 Department of Electrical and Computer Engineering, University of Utah, Salt Lake City, UT 84112 USA; 2 Department of Chemical Engineering, University of Utah, Salt Lake City, UT 84112 USA

**Keywords:** Graphene, Metamaterials

## Abstract

Optoelectronic devices in the UV range have many applications including deep-UV communications, UV photodetectors, UV spectroscopy, etc. Graphene has unique exciton resonances, that have demonstrated large photosensitivity across the UV spectrum. Enhancing UV absorption in graphene has the potential to boost the performance of the various opto-electronic devices. Here we report numerical study of UV absorption in graphene on aluminum and magnesium hole-arrays. The absorption in a single-layer graphene on aluminum and magnesium hole-arrays reached a maximum value of 28% and 30% respectively, and the absorption peak is tunable from the UV to the visible range. The proposed graphene hybrid structure does not require graphene to be sandwiched between different material layers and thus is easy to fabricate and allows graphene to interact with its surroundings.

## Introduction

The remarkable optical, electronic and mechanical properties of graphene have drawn much interest in the research community since its discovery in 2000^1–3^. Examples of graphene-based devices include high speed transistors, flexible electronics, high-speed electro-absorption modulators, transparent electrodes, photodetectors, molecule sensors, etc^4–7^. Enhancement of the light-matter interaction is crucial in boosting the performance metrics of the various optical devices based on graphene. Surface plasmon resonance (SPR) dramatically enhances the local electric fields and has been used to enhance the performance of graphene coupled devices in the terahertz (THz) and infra-red range (IR)^8,9^. Other examples of SPR enhanced graphene devices include photon detectors, gas sensors, and DNA sensors^10,11^.

Comparatively, when considering graphene devices, less attention has been focused in the ultraviolet (UV) region than in other regions of the electromagnetic spectrum. Opto-electronic devices in the UV range can be used for deep-UV communications, UV photodetectors, UV spectroscopy, etc^12–14^. In terms of biosensing, absorption and native fluorescence of biomolecules resides in the UV range. Therefore, enhancing UV absorption in graphene has the potential to boost the performance of these optoelectronic and biosensing devices. In the UV range, the exciton resonances at the saddle point in the band structure of graphene produce an abnormal absorption peak near 4.5 eV. The position and the shape of the exciton peak is a strong function of the free charge carrier density in graphene^15^, due to the screening effect of the many-body interactions at the saddle point. These unique exciton resonances have demonstrated large photosensitivity across the UV spectrum^16^. Graphene has been shown to boost the performance of photosensitivity of UV detectors, and enhance UV photoluminescence^17,18^. However, in-depth analysis of UV absorption in graphene is still at an early stage.

Several research articles have studied enhancing UV absorption in graphene^19–24^. All-dielectric absorbers have been proposed for perfect UV absorption in a single-layer graphene^19^. Metal-dielectric-metal plasmonic structures achieved numerically enhanced absorption of UV light in a single-layer graphene up to 50%^23^. Almost all investigated structures consist of a graphene layer sandwiched between a top dielectric or metal layer and a substrate that acts like a mirror. In practice, fabricating such sandwiched structures can be challenging since the fabrication steps associated with the top layers can alter the physical properties of graphene. Besides, having graphene embedded in between materials layers will limit its interactions with the environment, which is fundamental for the purpose e.g. of sensing applications. Here through FDTD simulations, we propose to use an aluminum and magnesium hole-array to enhance UV absorption in a single-layer graphene. These structures are easy to fabricate in practice; graphene can be transferred to the hole-arrays without additional deposition/lithography steps. The metals of the hole-arrays are aluminum or magnesium. These metals are chosen due to their excellent plasmonic properties in the UV range of the spectrum^25,26^. The graphene on aluminum and magnesium hole-arrays achieved 28% and 30% absorption at 300 nm, respectively, and the absorption peak is tunable from the UV to the visible range.

## Methods

**Figure 1:**
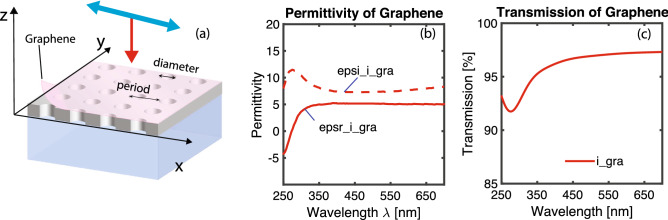
(**a**) A schematic diagram showing graphene on top of a metallic hole-array. (**b**) Real and imaginary permittivity of an intrinsic graphene. (**c**) Transmission spectrum through a free-standing intrinsic graphene.

Numerical simulations of the transmission spectrum of an aluminum or magnesium hole-array with graphene were carried out using finite-difference time-domain method (FDTD Solutions, Lumerical). As shown in Fig. 1a, circular air hole arrays (diameter 130 nm) were formed in a 100 nm aluminum or magnesium film on a 5 μm thick quartz substrate. A diameter of 130 nm was chosen to ensure that the holes are small enough (less than $\lambda$/2, where $\lambda$ $\sim$ 270 nm is the absorption peak of a free-standing intrinsic graphene), so that they do not support any propagating modes^27^, but large enough to exhibit large transmittance. Since no propagating modes are supported, the enhanced transmittance observed through the hole-array is associated with an excitation of plasmonic resonances on the front and back of the metallic film. One single-layer graphene covered the aluminum or magnesium hole-array and in the hole regions was immersed/suspended in air. A single unit cell of the array with graphene is modeled under an incident plane wave with transverse magnetic (TM) polarization towards the -z direction, and periodic boundary conditions used along x and y directions(Fig. 1a). Perfectly matched layer (PML) boundaries were set along ± z direction to absorb light waves with minimal reflections at the simulation edge. A z-normal Fourier transform (DFT) monitor sheet was placed below the hole array to collect the transmission data as a function of wavelength. The aluminum film was chosen to be 100 nm to be optically thick, so transmission is only possible through surface plasmon enhanced optical transmission. The dielectric constant data for aluminum was taken from the CRC Handbook of Chemistry and Physics^28^. And the dielectric constant for Al_2_O_3_ was taken from the Handbook of Optical Constants of Solids by Edward D. Palik^29^. The dielectric constant data for magnesium and magnesium oxide (MgO) was taken from the literature^30^. Since aluminum form an oxide layer at the interface between aluminum and air, an Al_2_O_3_ layer with 5 nm thickness was added at the interface between the aluminum film and air. In addition, an Al_2_O_3_ layer with 4 nm thickness was inserted at the interface between the aluminum film and the quartz substrate. As for magnesium, a MgO layer with 5 nm thickness was added at the interface between the magnesium film and air. In addition, a MgO layer with 4 nm thickness was inserted at the interface between the aluminum film and the quartz substrate. The thicknesses of oxide layers are taken from our previous studies^25^.

The optical constants of an intrinsic single-layer graphene were taken from the literature^31^. Figure 1b plots the permittivity of a single-layer graphene. The complex conductivity of graphene $\sigma$ was calculated from the optical constant data^32^ and imported into Lumerical as the 2-dimensional material model for a single-layer graphene. In order to reduce the computation volume to a manageable size, the thickness of graphene layer in the 2-dimensional model was chosen to be 2 nm instead of 0.34 nm (the thickness of an atomic layer). The complex conductivity employed to model the 2 nm graphene is calculated by multiplying $\sigma$ by the ratio of 0.34 nm/2 nm. Employing real and imaginary permittivity for an intrinsic layer of graphene as depicted in Fig. 1b, the transmission spectrum through a graphene sheet (without hole-arrays) was simulated and is plotted in Fig. 1c. The transmission spectrum for a single-layer graphene (Fig. 1c), from these simulations, matches well with the data from the literature. The absorption in a single-layer graphene in the visible range is close to $\pi \alpha$ (around 2.3%), where $\alpha =e^2/\hbar c \approx 1/137$ is the fine structure constant, consistent with what have been observed in the literature^33,34^. The excellent agreement between the simulated transmission spectrum and the theoretical one validates the accuracy of the graphene model that was used in the simulations.

Convergence tests on the thicknesses of the graphene model and mesh sizes were carried out. The differences of the transmission intensity through a 2 nm and 1 nm thickness graphene model was found to be less than 0.1%. Thus, the thickness of the graphene layer was set to 2 nm in the simulation to achieve an optimal balance between the simulation accuracy and the simulation size. An overall mesh of 2 nm was used to cover the hole-array and graphene layer and an additional mesh of 1 nm was used to cover just the graphene region. Convergence test on different mesh sizes on the graphene layer was also conducted by varying mesh size from 4 to 0.5 nm and less than 0.25% deviation was found below mesh size 1 nm (see Fig. S1 in supplementary information). Therefore, 2-dimensional graphene was modeled with a 2 nm thickness and a 1 nm mesh size was used in the simulation.

## Results

Aluminum and magnesium hole-arrays exhibit extraordinary transmission in the UV and visible range of the electromagnetic spectrum^25^. The resonances of aluminum and magnesium hole-arrays are tunable depending on the periodicity of the hole-arrays. We employ an aluminum and magnesium hole-array with SPR resonances in the UV range to couple with the graphene excitonic resonances. The localized near fields with enhanced in-plane components will drastically enhance the absorption in graphene. We simulated a single-layer graphene placed directly on top of an aluminum and magnesium hole-array with periodicity from 200 to 400 nm.

**Figure 2:**
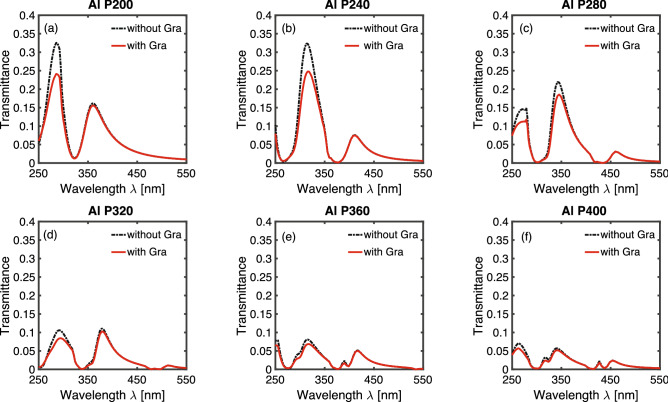
Transmission spectrum of an aluminum hole-array with and without graphene. The periodicities of the aluminum hole-arrays are (**a**) 200 nm (**b**) 240 nm (**c**) 280 nm (**d**) 320 nm (**e**) 360 nm (**f**) 400 nm. The dashed black curve represents the spectrum for an aluminum hole-array without graphene layer; the solid red line corresponds to the spectrum for an aluminum hole-array with an intrinsic graphene layer.

Figure 2a–f plot the transmission spectrum of an aluminum hole-array with periodicity from 200 to 400 nm in 40 nm increment. There are two curves in each figure -the dashed black curve is the spectrum for just an aluminum hole-array without graphene layer; the solid red line is the spectrum for an aluminum hole-array with an intrinsic graphene layer. Multiple transmission peaks and dips occur in the transmission spectrum that red-shift with increasing periodicities. The positions of transmission peaks and dips remain unchanged after addition of a single-layer graphene, while the intensity of transmission peaks is reduced. At longer wavelength in the visible range, the spectrum for hole-array with and without graphene almost overlaps for all periodicities. This is due to the fact that at visible wavelengths, the graphene absorption is very small, at around 2.3% independent of wavelength. At wavelengths in the ultraviolet range (less than 400 nm), the transmission intensity with graphene is reduced due to the strong absorption of graphene in the UV range.

**Figure 3:**
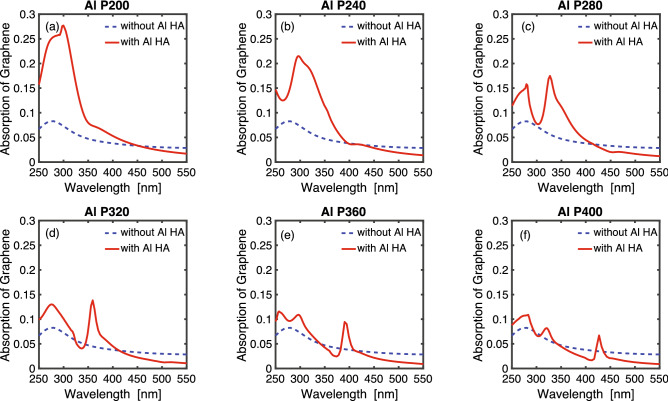
Graphene absorption spectrum on an aluminum hole-array. The periodicities of the aluminum hole-array are (**a**) 200 nm (**b**) 240 nm (**c**) 280 nm (**d**) 320 nm (**e**) 360 nm (**f**) 400 nm. The solid red line represents the absorption in graphene on an aluminum hole-array. The dashed blue line represents the absorption in an intrinsic monolayer of graphene without hole-arrays.

Figure 3 plots the absorption in graphene suspended in air (dashed blue line), versus that in graphene on top of an aluminum hole-arrays with different periodicities (solid red line). The absorption in graphene is calculated by using the formula Eq. 1 below^22^, where $E_{l}$ is the in-plane component of the electric field. $\left| E_{l} \right| ^{2}=\left| E_{x} \right| ^{2}+\left| E_{y} \right| ^{2}$. The out-of-plane component $E_{n}$ does not contribute to the graphene absorption due to the anisotropic dispersive dielectric constant of graphene^22^. In Eq. 1, *N* is the complex optical constants of the graphene. The absorption in graphene alone has a maximum value around 10% near 270 nm, while the absorption in graphene on top of a hole-array has a tunable peak that is dependent on the hole-array spacing. For aluminum hole-arrays with large periodicities, there are multiple absorption peaks that correspond to aluminum hole-array SPR resonance peaks. It is observed that the graphene absorption peak red-shifts with increasing periodicity and the absorption intensity also reduces with increasing periodicity. The highest absorption 28% happens for aluminum hole-array with periodicity 200 nm at a wavelength of 300 nm, which is nearly $\sim 3$ times larger than that in graphene alone. The enhanced graphene absorption originates from the strong localized in-plane electric field components generated by UV SPR at the graphene. At longer wavelengths ($\lambda >400$ nm), the absorption in graphene alone is higher than that in graphene on hole-arrays. This is also observed at transmission dip positions at shorter wavelengths. The reduced absorption in graphene on aluminum hole-arrays is caused by the rotation of the Poynting vector at the edge of the holes, which resulted in a reduction in the in-plane components of the electric fields^25^.

1 $$\begin{aligned} A(\lambda )=-\frac{2\pi \cdot c}{\lambda }\cdot Re(N)\cdot Im(N)\cdot \int _{v}\left| E_{l} \right| ^{2}dV \end{aligned}$$

**Figure 4:**
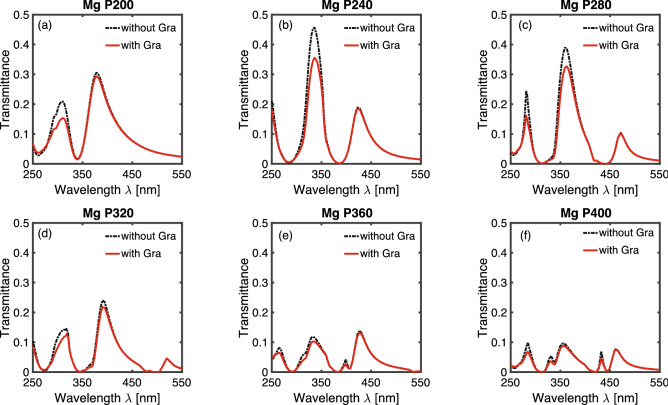
Transmission spectrum of a magnesium hole-array with and without a single-layer intrinsic graphene. The periodicities of the magnesium hole-arrays are (**a**) 200 nm (**b**) 240 nm (**c**) 280 nm (**d**) 320 nm (**e**) 360 nm (**f**) 400 nm. The dashed black curve represents the spectrum for a magnesium hole-array without graphene layer; the solid red line represents the spectrum for a magnesium hole-array with graphene layer.

In addition to aluminum hole-arrays, we also simulated graphene absorption on magnesium hole-arrays. The SPR figure of merit for magnesium has similar amplitude compared to that for aluminum in the UV range^25^. Figure 4a–f depict the transmission spectrum of a magnesium hole-array with periodicity from 200 to 400 nm in 40 nm increment. There are two curves in each figure: the dashed black curve represents the spectrum for just a magnesium hole-array without graphene layer; the solid red line represents the spectrum for a magnesium hole-array with an intrinsic graphene layer. We noticed similar features in the spectrum than those observed with aluminum hole-arrays. First, the spectrum overlaps for all periodicities at longer wavelengths in the visible range. At wavelength in the ultraviolet range (less than 400 nm), the transmission intensity is reduced once a single-layer graphene is added to the hole-array. In comparison with aluminum hole-arrays, the transmission peaks and dips in magnesium hole-arrays red-shift about 20 nm. In addition, the transmission intensity for magnesium hole-arrays is larger compared with aluminum hole-arrays. These characteristics are consistent with prior studies on transmission spectrum on aluminum and magnesium hole-arrays^25^. The elevated transmission intensity of magnesium hole-arrays, compared with aluminum hole-arrays is consistent with the larger graphene absorption on magnesium hole-arrays, as will be discussed below.

**Figure 5:**
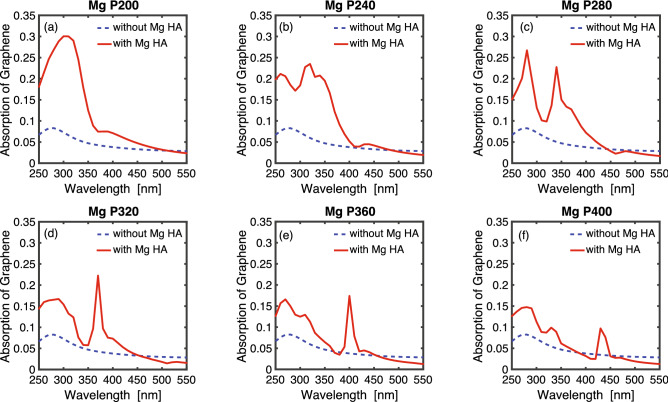
Graphene absorption spectrum on a magnesium hole-array. The periodicities of the magnesium hole-array are (**a**) 200 nm (**b**) 240 nm (**c**) 280 nm (**d**) 320 nm (**e**) 360 nm (**f**) 400 nm. The solid red line represents the absorption in graphene on a magnesium hole-array. The dashed blue line represents the absorption in an intrinsic monolayer graphene without hole-arrays.

Figure 5 plots the absorption intensity in the graphene layer without magnesium hole-arrays (dashed blue line), versus the absorption intensity in the graphene layer with magnesium hole-arrays for different periodicities (solid red line). It is observed that the graphene absorption peak red-shifts with increasing periodicity and the absorption intensity also reduces with increasing periodicity. The highest absorption 30% happens for magnesium hole-array with 200 nm periodicity at a wavelength of 300 nm. For the same periodicity, graphene absorption is higher on magnesium hole-arrays compared with aluminum hole-arrays due to stronger near field intensity sustained by magnesium hole-arrays. This stronger near field is manifested by elevated transmission intensity in Fig. 4.

**Figure 6:**
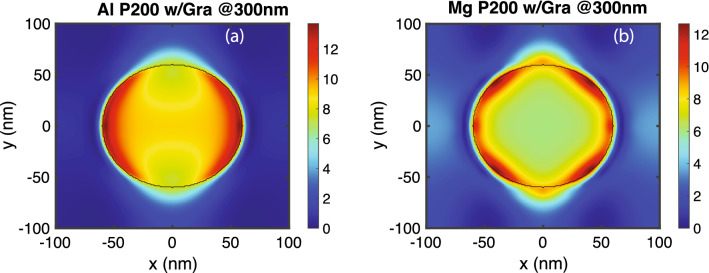
In-plane components of the electric field in graphene at the maximum absorption wavelength. (**a**) $\left| E_{l} \right| ^{2}$ in a monolayer of graphene on an aluminum hole-array with periodicity 200 nm at $\lambda =300$ nm. (**b**) $\left| E_{l} \right| ^{2}$ in a monolayer of graphene on a magnesium hole-array with periodicity 200 nm at $\lambda =300$ nm.

The enhanced graphene absorption is due to the enhanced $\left| E_{l} \right| ^{2}$ at the interface of metal and air on plasmonic hole-arrays. To visualize the pattern of $\left| E_{l} \right| ^{2}$, Fig. 6 plots $\left| E_{l} \right| ^{2}$ inside graphene at the maximum absorption wavelength for aluminum and magnesium hole-arrays. Figure 6a plots $\left| E_{l} \right| ^{2}$ on aluminum hole-array with periodicity 200 nm at a wavelength of 300 nm. Figure 6b plots $\left| E_{l} \right| ^{2}$ on magnesium hole-array with periodicity 200 nm at a wavelength of 300 nm. The field is mostly concentrated at the edge of the hole with orientation in parallel with the polarization of the light.

**Figure 7:**
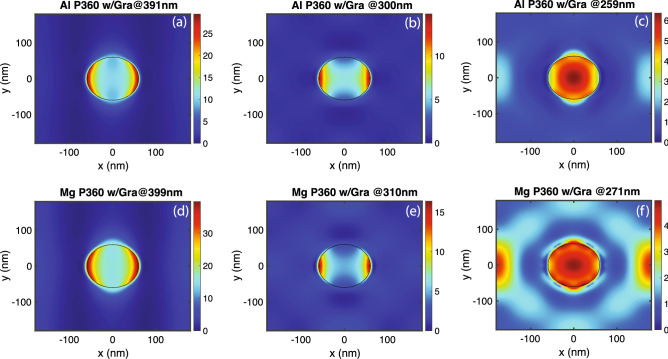
In-plane components of the electric field in graphene at multiple absorption peak positions. (**a**) $\left| E_{l} \right| ^{2}$ in a monolayer of graphene on an aluminum hole-array with periodicity 360 nm at $\lambda =391$ nm. (**b**) $\left| E_{l} \right| ^{2}$ in a monolayer of graphene on an aluminum hole-array with periodicity 360 nm at $\lambda =300$ nm. (**c**) $\left| E_{l} \right| ^{2}$ in a monolayer of graphene on an aluminum hole-array with periodicity 360 nm at $\lambda =259$ nm. (**d**) $\left| E_{l} \right| ^{2}$ in a monolayer of graphene on a magnesium hole-array with periodicity 360 nm at $\lambda$= 399 nm. (**e**) $\left| E_{l} \right| ^{2}$ in a monolayer of graphene on a magnesium hole-array with periodicity 360 nm at $\lambda$=310 nm. (**f**) $\left| E_{l} \right| ^{2}$ in a monolayer of graphene on a magnesium hole-array with periodicity 360 nm at $\lambda =271$ nm.

For aluminum and magnesium hole-arrays with periodicity 200 nm, there is only one graphene absorption peak, which corresponds to the first order air-metal SPR mode. For hole-arrays with large periodicity (> 200 nm for Mg and >240 nm for Al), the first order air-metal SPR mode red-shifts to longer wavelengths, while multiple absorption peaks emerge at shorter wavelengths. For example, for aluminum hole-arrays with periodicity 360 nm, there are three absorption peaks at around 390 nm, 300 nm, 260 nm. The first order air-metal SPR mode corresponds to the absorption peak around 390 nm, while higher order SPR modes correspond to absorption peaks at shorter wavelengths. Figure 7a–c plot $\left| E_{l} \right| ^{2}$ inside graphene at the three peak wavelengths on the aluminum hole-array with periodicity 360 nm. It is observed that intensity of $\left| E_{l} \right| ^{2}$ reduces for higher order modes, while extends further inside of the holes. Despite the reduction in $\left| E_{l} \right| ^{2}$, the graphene absorption peak at shorter wavelength (higher order modes) is comparable to that at longer wavelength due to the extended high field regions. Similar pattern can be observed for the Mg 200 nm hole-array in Fig. 7d–f.

## Conclusion

Enhancing graphene absorption in the ultraviolet range can boost the performance of optoelectronic and biosensing devices. Here we demonstrated numerically that by coupling graphene with aluminum and magnesium hole-arrays, up to 30% absorption in graphene at a wavelength of 300 nm can be achieved. By tuning the periodicities of the hole-arrays, the graphene absorption peaks can be shifted towards the visible range. The positions of the graphene absorption peaks correspond to the UV SPR modes on the hole-arrays. Multiple graphene absorption peaks are found for hole-arrays with large periodicities. The proposed structures are easy to fabricate without additional lithography steps on top of graphene.

## Supplementary Information

Supplementary Information.

## Data Availability

The datasets generated during and/or analyzed during the current study are available from the corresponding author on reasonable request.
