# Supplementary material for: Enhanced ultraviolet absorption in graphene by aluminum and magnesium hole-arrays
Source: Sci Rep. 2021 Apr 19;11:8516. doi: 10.1038/s41598-021-87868-7 (PMC8055649; doi:10.1038/s41598-021-87868-7)
Supplement: Supplementary file 1 — Supplementary Information. [file 41598_2021_87868_MOESM1_ESM.pdf]

Supplementary information for Enhanced ultraviolet absorption in graphene by aluminum and magnesium hole-arrays

XUELING CHENG,[1] AND YUNSHAN WANG[1,2*]

[1]*Department of Electrical and Computer Engineering, University of Utah , Salt Lake City, UT 84112, USA*
[2]*Department of Chemical Engineering, University of Utah, Salt Lake City, UT 84112, USA*
[*]*yunshan.wang@utah.edu*

Convergence test on different mesh sizes on the graphene layer was conducted by varying mesh size from 4nm to 0.5nm. The Error in percentage versus mesh size is plotted in Fig. S1.

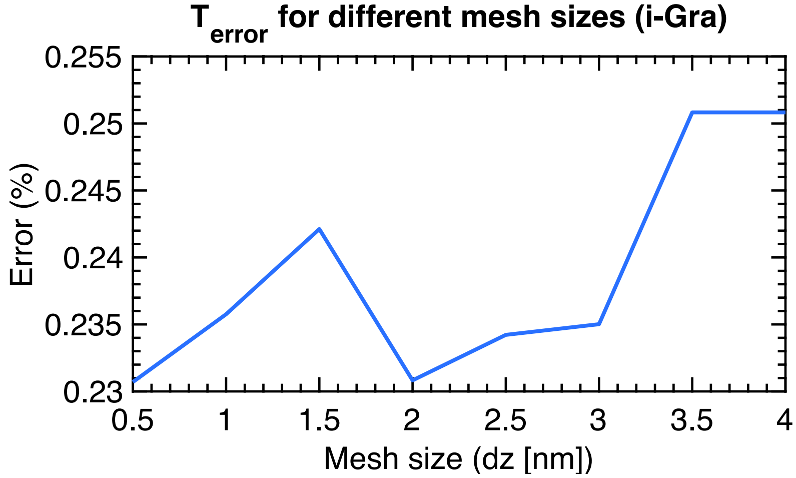

Figure S1: Convergence test on different mesh sizes on the graphene layer.

$$T_{error} = \sqrt{\frac{\int_{240nm}^{700nm} (T_{simulated}(\lambda) - T_{literature}(\lambda))^2 d\lambda}{\int_{240nm}^{700nm} (T_{literature})^2 \, d\lambda}}$$

T_{error} is the error for a certain mesh size in FDTD simulation.

$T_{simulated}$ is wavelength dependent transmission of single layer intrinsic graphene from FDTD simulation for a certain mesh size.

$T_{literature}$ is wavelength dependent transmission of single layer intrinsic graphene from literature.[1]

[1] V. Kravets, A. Grigorenko, R. Nair, P. Blake, S. Anissimova, K. Novoselov, and A. Geim, “Spectroscopic ellipsometryof graphene and an exciton-shifted van hove peak in absorption,” Phys. Rev. B81, 155413 (2010)
